# A Reflection on the Tensions of Acting in Dual Roles of Doctoral Researcher and Practitioner when Evaluating a Lifestyle Intervention for Breast Cancer Patients

**DOI:** 10.1007/s13187-022-02180-w

**Published:** 2022-05-23

**Authors:** Jane Richardson, Rosie Erol, Allain Amador Bueno

**Affiliations:** 1 School of Allied Health and Community, University of Worcester, Henwick Grove, Worcester, WR2 6AJ UK; 2 School of Psychology, University of Worcester, Henwick Grove, Worcester, WR2 6AJ UK; 3 School of Science and the Environment, University of Worcester, Henwick Grove, Worcester, WR2 6AJ UK

**Keywords:** Reflective research, Dual role research, Lifestyle intervention evaluation, Research ethics

## Abstract

This reflection was completed as part of a doctoral project to develop and trial a lifestyle intervention for people following the completion of their treatment for breast cancer. In this study the graduate student acted in the dual roles of nutrition practitioner and researcher. This article uses the experience, reflection, action (ERA) cycle of reflection to consider some of the tensions faced due to the divergent priorities and requirements of these two roles. One challenge occurred during study recruitment when a few potential participants did not meet the inclusion criteria for the study but still wished to attend the intervention sessions. It was also a challenge to mitigate the risks of distress of potentially vulnerable participants during group intervention sessions. In both instances there was a potential conflict between the needs of patients and research requirements. This reflection concluded that the obligations of both roles should be adhered to where possible, but if in doubt, the needs of the participants were paramount.

## Introduction

I was a lecturer in nutrition when I began my PhD, and for a few years had been part of a team delivering a lifestyle programme for local people after their breast cancer treatment. I wanted to evaluate and improve this programme during my doctoral study [1]. During this project, I continued to lead the intervention and ran the nutrition sessions, but I was also the researcher. I was unsure which of these roles should take precedence. I have reflected on some of the challenges that I faced in relation to these dual roles using the experience, reflection, action (ERA) cycle of reflection [2] to guide my thinking and writing.

## Experience

I experienced tension between my roles of practitioner and researcher at two points in my study. The first time this happened was while I was recruiting participants. People who were interested in joining my study contacted me and I spoke to them on the phone and sent them further information to read. If they were still interested, I arranged to meet them to answer any questions, check their details, and go through what would happen next. I was really surprised in these meetings to find that a few people were still in the final stages of their radiotherapy treatment. This meant that they did not meet one of the inclusion criteria for my study, but they were still really keen to join the lifestyle programme. As a researcher I knew that these potential participants should be excluded from the research. However, as a practitioner I wanted these participants to have the choice to attend the intervention sessions if they wished to.

I experienced another tension between my two roles during one particular intervention session. We discussed some booklets with lifestyle advice to reduce cancer risk; the same advice was also recommended for cancer survivors. These recommendations evoked anger and distress in some participants. As a practitioner I wanted to alter future sessions to make sure that this did not happen again, while as a researcher I wanted to make sure that the sessions stayed the same to maintain the fidelity of the intervention.

I was unsure what to do in both of these cases. I discussed the issues with my PhD supervisors which helped me to reflect and find a way forward.

## Reflection

The potential participants who were still in treatment wanted to join the programme to get support for their transition from being a patient back to a normal healthy life. My own values as a practitioner meant that I did not want to exclude them as they might benefit from attending. I was aware that some other studies have found that recruitment during breast cancer treatment might be feasible and beneficial [3, 4]. However, these potential participants did not meet all of the inclusion criteria in my research protocol. This situation had occurred while I was recruiting for my final two intervention groups. Therefore, it was too late for me to apply for approval to amend my inclusion criteria as my study had already begun, and an initial participant group had been recruited and had attended their group sessions. Neither could I offer these participants a later intervention group as it was not clear if one would be available. A similar tension in the practitioner-researcher role in recruitment was explored in another study in which a randomised controlled trial (RCT) of a healthy lifestyle intervention for people with mental health diagnoses was carried out [5]. In this study by Park et al., some of those allocated to the parallel control group insisted on attending the intervention sessions intended for the trial group. They were not prepared to wait to attend a later intervention group after the RCT trial was completed. The nurse researchers in this study reflected on this in the light of their dual roles and decided that the participant needs were paramount [5]. In their study these control participants were therefore not excluded from attending the intervention sessions, with a concomitant impact on their study data and outcomes [5].

My own study was an example of applied research and I aimed to evaluate our lifestyle intervention to improve it to better meet people’s needs. It seemed to me that it would be counter to my aim to exclude people against their wishes. I had designed my study with a participatory and co-operative view of research, and I recruited people as participants, not research subjects. Therefore, I regarded the views and needs of participants as important, and this was consistent with my values as practitioner and researcher. My study design was also influenced by my values and by my background as a lecturer. I opted for a quasi-experimental design [6] in which participants acted as their own controls. I did this to avoid recruiting participants who would be allocated to a control group, so I was able to avoid the challenge encountered in the RCT discussed above [5].

Due to my previous experience of running lifestyle interventions, I was aware that people joining my study might be considered to be a “vulnerable” population. There are many different meanings of this term; for example, in the context of seeking consent for a research study, the University of Worcester ethics policy [7] lists vulnerable groups as children, persons lacking mental capacity, and persons whose first language is not English. The participants in my study were not vulnerable in these terms. However**,** cancer survivors, in general, can be considered to be a vulnerable population in terms of having ongoing health problems, reduced quality of life, and increased psychological distress [8–11]. A contrasting view is that all humans are vulnerable to some extent, depending on context [12]. During my first meetings with participants, some people identified that they were still coming to terms with their diagnosis and felt anger or fear, while other participants also had to face further treatment such as reconstructive surgery. I was also aware that people who have had breast cancer may experience strong emotions due to the trauma of diagnosis, fears of recurrence, experience of family distress, and greater awareness of their own mortality [10, 13]. For some, this can lead to an increased risk of depression, sleep disturbance, sadness, reduced quality of life, and cognitive dysfunction [11]. I was aware that participants’ emotions might be stirred during group sessions as for some this might have been their first opportunity to discuss their experiences with others in a similar position. I was aware that discussions of lifestyle improvements might provoke negative emotions about their previous lifestyle, and I attempted to plan the intervention accordingly to mitigate those risks. I used booklets as discussion prompts and checked that these were evidence based and relevant for the group. However, some people became upset when reviewing these publications about lifestyle recommendations to reduce cancer risk and for cancer survivors [14]. Some participants were angry as they had previously had a healthy lifestyle and yet had still developed breast cancer, while others felt that the recommendations implied that they were to blame for their own diagnosis, or that it was too late to reduce their risks as they had already had a cancer diagnosis. As a practitioner, I really wanted to reflect on this and amend the sessions to avoid causing distress in any future similar sessions. However as a researcher, I was also aware that fidelity of intervention was important to my study outcomes [15] and so I should, as far as possible, keep my sessions the same for each group.

Role conflict in health research is often a conflict between patient needs and research requirements [16, 17]. Hay-Smith and colleagues suggest that clinician researchers should prioritise both patient wellbeing and the requirements of ethical research [17], and this was the joint approach that I tried to take. Following my reflection on the tensions that I experienced between my dual roles of researcher and practitioner, I decided that I would try to adhere to both sets of obligations where possible, but if in doubt, the needs of the participants were paramount.

## Action

I decided that although potential participants could not join the research study until their treatment was completed in line with my ethical approval, they were still able to join the group sessions if they wished. I invited them to join the sessions with a full understanding that they would not be able to provide research data until after their treatment period. I was able to include these additional people without preventing anyone who was eligible for the research study from joining, as the intervention was not oversubscribed. The negative reactions to our discussion of lifestyle recommendations only occurred once with the first intervention group, and I reflected on the issues before I repeated the session. With subsequent groups I shifted the emphasis and focused our discussions of the lifestyle recommendations more on the benefits for cancer survivors rather than on reducing cancer risk. I discussed the same recommendations using the same resources with subsequent groups, with no further apparent upset. Therefore, I was able to maintain intervention fidelity. Following my reflections, I recommend that future lifestyle interventions focus more on wellbeing and recovery than cancer risk and that interventions might be more beneficial for those who are ready to make behaviour changes, whether or not they are still completing treatment. In both cases, these actions allowed me to follow my obligations both as a researcher and as a practitioner while remaining true to my own values.
